# Severe gastrointestinal injury associated with SARS-CoV-2 infection: Thrombosis or Inflammation?: A retrospective case series study

**DOI:** 10.1097/MD.0000000000031188

**Published:** 2022-10-21

**Authors:** Henry Robayo-Amortegui, Alex Forero-Delgadillo, Michel Pérez-Garzón, Claudia Poveda-Henao, Conny Muñoz-Claros, Andrea Bayona-Solano, Carlos Orozco, Ricardo Buitrago-Bernal

**Affiliations:** a Universidad de La Sabana, Chía, Colombia; b Intensive Care, Fundación Clínica Shaio, Bogotá, Colombia; c Pathologist, Clínica Shaio, Bogotá, Colombia.

**Keywords:** acute gastrointestinal injury, bowel ischemia, bowel perforation, COVID-19, critically ill patients

## Abstract

Acute gastrointestinal injury (AGI) associated with severe acute respiratory syndrome coronavirus 2 (SARS-CoV-2) infection has a low incidence of complications in patients admitted to the intensive care unit (ICU). Pathophysiological knowledge related to AGI is limited, as few studies have been published on this topic. Therefore, this study was carried out to identify the clinical and histopathological features of patients with SARS-CoV-2 infection and grade IV AGI.

**Methods:**

This is a retrospective case study of fifteen patients with SARS-CoV-2 infection and grade IV AGI who underwent emergency surgery.

**Results:**

This study revealed a mortality rate of 62.5%. The most frequent gastrointestinal symptoms were abdominal distension (100%) and increased gastric residual volume (93.3%). Distended bowel loops on plain abdominal radiography (90%) and intestinal pneumatosis on computed tomography (50%) were the most frequent imaging findings. Surgical exploration revealed intestinal ischemia (66.6%) and necrosis (46.6%), and histopathology showed ischemic and liquefactive necrosis with mixed inflammatory involvement and absence of thrombosis as the cause of AGI.

**Conclusions:**

AGI associated with severe SARS-CoV-2 infection has a high mortality rate and poses a diagnostic challenge in the ICU. The complex pathophysiology and histopathological findings indicate an associated inflammatory phenomenon as the main alteration in the absence of thrombosis, as per the intestinal biopsies of the cases studied. Further clinical studies are required to gain a better understanding of this pathology.

## 1. Introduction

The coronavirus disease 2019 (COVID-19) pandemic has spread worldwide, with more than 291 million confirmed cases and approximately 5 million deaths to date.^[1]^ Between 14% and 20% of patients require hospitalization, of which approximately 2% require admission to the intensive care unit (ICU). The mortality rate in critically ill patients is approximately 60%. Furthermore, 30% of these deaths are caused by extrapulmonary complications.^[1,2]^ In critically ill patients, acute gastrointestinal injury (AGI) that is not related to severe acute respiratory syndrome coronavirus 2 (SARS-CoV-2) infection has been found to have a prevalence rate of 40%, which nearly doubles the mortality rate compared to that in patients without this complication.^[3]^ Since 2012, the Working Group on Abdominal Problems of the European Society for Intensive Care Medicine has classified AGI into grades I to IV, the latter being related to progression associated with multiple organ dysfunction syndrome (MODS) and subsequent death.^[4]^

AGI is not frequently observed in patients with severe SARS-CoV-2 infection; however, when it occurs, the mortality rate can reach 58%.^[5]^ Currently, there are few publications on severe SARS-CoV-2 infections associated with severe AGI (Grade IV). In this case series, we describe 15 patients with severe pneumonia due to COVID-19 who were managed in the ICU, presented with grade IV AGI as a complication, and required emergency surgery.

## 2. Methodology

Data were collected from the electronic medical records of patients admitted to a fourth-level institution in Bogotá, Colombia, between October 1, 2020 and July 31, 2021. The study was approved by the local ethics committee (Comité de Ética Fundación Clínica Shaio) under number DIB-20-42, the ethics committee waived the need for informed consent considering the retrospective nature of data collected. This study included patients aged > 18 years with SARS-CoV-2 infection confirmed by a COVID-19 reverse transcription polymerase chain reaction test who had to be admitted to the ICU and presented with grade IV AGI complications, which were defined as marked gastrointestinal insufficiency with severe organ (pulmonary and cardiovascular) involvement, intolerance to nutrition, diarrhea or ileus, upper or lower gastrointestinal bleeding, mesenteric ischemia, or Ogilvie syndrome requiring emergency laparotomy during their stay. The sample was analyzed using descriptive statistics. For continuous variables, measures of central tendency and dispersion were calculated, whereas for qualitative variables, distributions of absolute and relative frequencies were estimated. Statistical graphs were created to illustrate the behavior of the variables.

## 3. Results

### 3.1. Population

Fifteen patients with grade IV AGI were included in this study. Of these, 67% were men with an average age of 55 years and an average body mass index of 30 kg/m^2^. Arterial hypertension and type II diabetes mellitus were the most common comorbidities (40%). The average number of days for which mechanical ventilation was provided was 15.3 days (standard deviation [SD]: 10 days) and the duration of stay in the ICU was 20.6 days (SD: 12.06 days). The overall mortality rate was 66% (Table 1).

**Table 1:** Demographic characteristics of critically ill patients with COVID-19 and AGI grade IV.

	All patients (N = 15)
**Age, mean (SD**)	55.07 (15.2)
**Male**	10 (66.7%)
**BMI, median (SD**)	30.0 (5.3)
**Type II diabetes mellitus**	6 (40%)
**Hypertension**	6 (40%)
**Vasopressor therapy**	13 (86.7%)
**Vasopressor use prior to GI symptoms**	2 (13.3%)
**ECMO**	2 (13.3%)
**Mechanical ventilation**	13 (86.7%)
**SOFA Score**	11.8 (3.4)
**Non-survivors**	10 (66.7%)
**Days ventilation mean (SD**)	15.27 (10)
**Days in ICU mean (SD**)	26.6 (12.06)

AGI = acute gastrointestinal injury, COVID-19 = coronavirus disease 2019, ECMO = extracorporeal membrane oxygenation, SD = standard deviation, SOFA = sequential organ failure assessment.

### 3.2. Clinical presentation

Patients with respiratory symptoms developed severe gastrointestinal symptoms after of 16 days (SD: 6.4 days); of these, 87% required vasopressor therapy. The main systemic manifestation was fever (in 40% of the cases), and the most frequent clinical findings were abdominal distension (100%), increased gastric residual volume (93%), diarrhea (40%), and constipation (33%) (Table 2).

**Table 2:** Symptoms, radiological and surgical findings of critically ill patients with COVID-19 and AGI grade IV.

	All patients (N = 15)	%
**GI manifestations during hospitalization**		
Fever before GI symptoms	9	60
Nausea	2	13.3
Jaundice	1	6.7
Diarrhea	6	40
Constipation	5	33.3
Abdominal pain	5	33.3
Abdominal distention	15	100
Gastric residue	14	93.3
**Abdominal X-ray**		
Pneumoperitoneum	2	13.3
Dilatation of small bowel loops	7	46.6
Dilatation of large bowel loops	5	33.3
Pneumatosis	1	6.6
**Simple and contrast abdominal tomography**		
Intestinal pneumatosis	5	33.3
Dilatation of small bowel loops	1	6.6
Intraperitoneal collection	3	20
Pneumoperitoneum	1	6.6
Intestinal ischemia	1	6.6
Intestinal perforation	1	6.6
**Surgical findings**		
Four quadrant peritonitis	9	60
Intestinal ischemia	10	66.6
Intestinal necrosis	7	46.6
Intestinal perforation	5	33.3
Open abdomen	12	80

AGI = acute gastrointestinal injury, COVID-19 = coronavirus disease 2019, GI = gastrointestinal injury.

### 3.3. Diagnostic imaging and anticoagulation therapy

Elevated D-dimer levels resulted in anticoagulant therapy in 93% of the patients. All the patients underwent diagnostic tests to investigate the presence of thromboembolic diseases. This study revealed deep vein thrombosis in 13% of patients and pulmonary embolism in 20% of patients despite treatment.

Plain abdominal radiography revealed dilatation of the small bowel loops in 47% of cases and pneumoperitoneum in 13.3%. Abdominal computed tomography with and without contrast was performed in 7 patients, all of whom showed abnormal findings. Pneumatosis was documented in 5 patients, followed by intraperitoneal fluid collection in 3 patients and intestinal ischemia, necrosis, and perforation in 1 patient (Table 2). Finally, the laboratory findings obtained before and after the surgical procedure are listed in Table 3.

**Table 3:** Laboratory findings of critical ill patients with COVID 19.

Median (IQR)			
	Range	24 h Pre-surgical	24 h Post-surgical
Leukocytes 10^3/mL	4.5 - 11	13.9 (8.4–32.7)	10.5 (6.65–19.1)
Lymphocyte 10^3/mL	1.5 - 4.0	8 (3–1.1)	7 (4 - 1.3)
Neutrophil 10^3/mL	1.5 - 8.0	12.4 (7.3–30.08)	7.9 (4.85 - 16.5)
Hemoglobin gr/dL	13.5 - 18	12.5 (11 - 16.2)	11.1 (9.6 -12.8)
Hematocrit %	40.0 - 54.0	37 (33 - 49)	34 (30.7 - 40.0)
Platelets mm^3^	150.000 - 450.000	334000(169.000 - 431.000)	240000 (9600 - 175.000)
D-dimer ng/mL	0 - 550	1505 (693 - 4.455)	10055 (9600 - 175.000)
Ferritin ng/mL	17 - 464	1027.5 (548 -1.485)	1850 (8300 - 10.055)
C-reactive protein mg/L	0 - 5	149.5 (52.75 - 368.75)	465 (270 - 660)
Lactate dehydrigenase U/L	120 - 246	455 (304 - 554)	1267.5 (420 - 1267)
Bicarbonate mEq/L	18 - 25.6	22 (18 - 26.2)	18 (15.7 - 23)
Lactate mmol/L	0.1 - 1.9	2.27 (1.7 - 3.2)	2.9 (1.49 -4.75)
Base excess mEq/L	-2 + 2	-4.8 (-8.4 - 1.8)	-5.4 (-10.6 - 2.7)
CO2 Delta mm Hg	6	5.2 (2.0 - 7.6)	5 (1.7 -7.6)
SvCO2 %	~70	71.5 (66.0 - 76.5)	67 (54.5 -78.5)
ETO2 %	30 -35	24.5 (20.8 - 28.8)	20 (14.5 - 29)
Oxygen Debt L/minO^2^	3.78	27.1	34.1
pH	7.35 - 7.45	7.28 (7.23 - 7.42)	7.3 (7.19 -7.43)
Sodium mEq/L	137 - 145	141 (138 - 145)	142 (137 - 148.2)
Potassium mMol/L	3.5 - 5.1	4.8 (3.8 - 5.4)	5.15 (4.7 -6.0)
Calcium mg/dL	8.8 - 10.2	7,95 (7,68 – 8,23)	7.1 (6.12 - 7.7)
Magnesium mg/dL	1.6 - 2.3	2.2 (2 - 2.4)	2.2 (2.1 -2.5)
Phosphorus mg/dL	2.5 - 4.5	4.8 (3.75 -7.65)	5.3 (2.25 -9.80)
Creatinine mg/dL	0.66 - 1.25	1.3 (0.7 - 3)	1.85 (0.77 - 4.3)
Urea nitrogen mg/dL	9.0 - 20	61 (23 -87)	61 (36.5 - 81.25)
Total bilirubin mg/dL	0.20 - 1.30	1 (0.65 - 2.02)	0.76 (0.61 - 1.46)
Delta-bilirubin mg/dL	0.00 - 0.20	0.7 (0.45 - 1.0)	0.6 (0.4 -0.90)
Indirect bilirubin mg/dL	0.00 - 1.10	0.41 (0.13 - 0.58)	0.2 (0.08 -0.52)
AST U/L	17 - 59	136.5 (65 - 357.2)	179 (56 - 374)
ALT U/L	0.0 - 50	87 (61.5 - 129)	110 (72 -180)

ALT = alanine aminotransferase, AST = aspartate aminotransferase, CO2 Delta = dioxide carbon delta, COVID-19 = coronavirus disease 2019, ETO2 = oxygen extraction fraction, IQR = interquartile range, SvCO2 = central venous oxygen saturation.

### 3.4. Surgical and histopathological findings

Intestinal ischemia and peritonitis in all 4 quadrants and intestinal necrosis were found in 67%, 60%, and 47% of the cases, respectively. Additionally, 80% of the patients required a second surgical intervention and 47% required right hemicolectomy.

**Figure 1.**
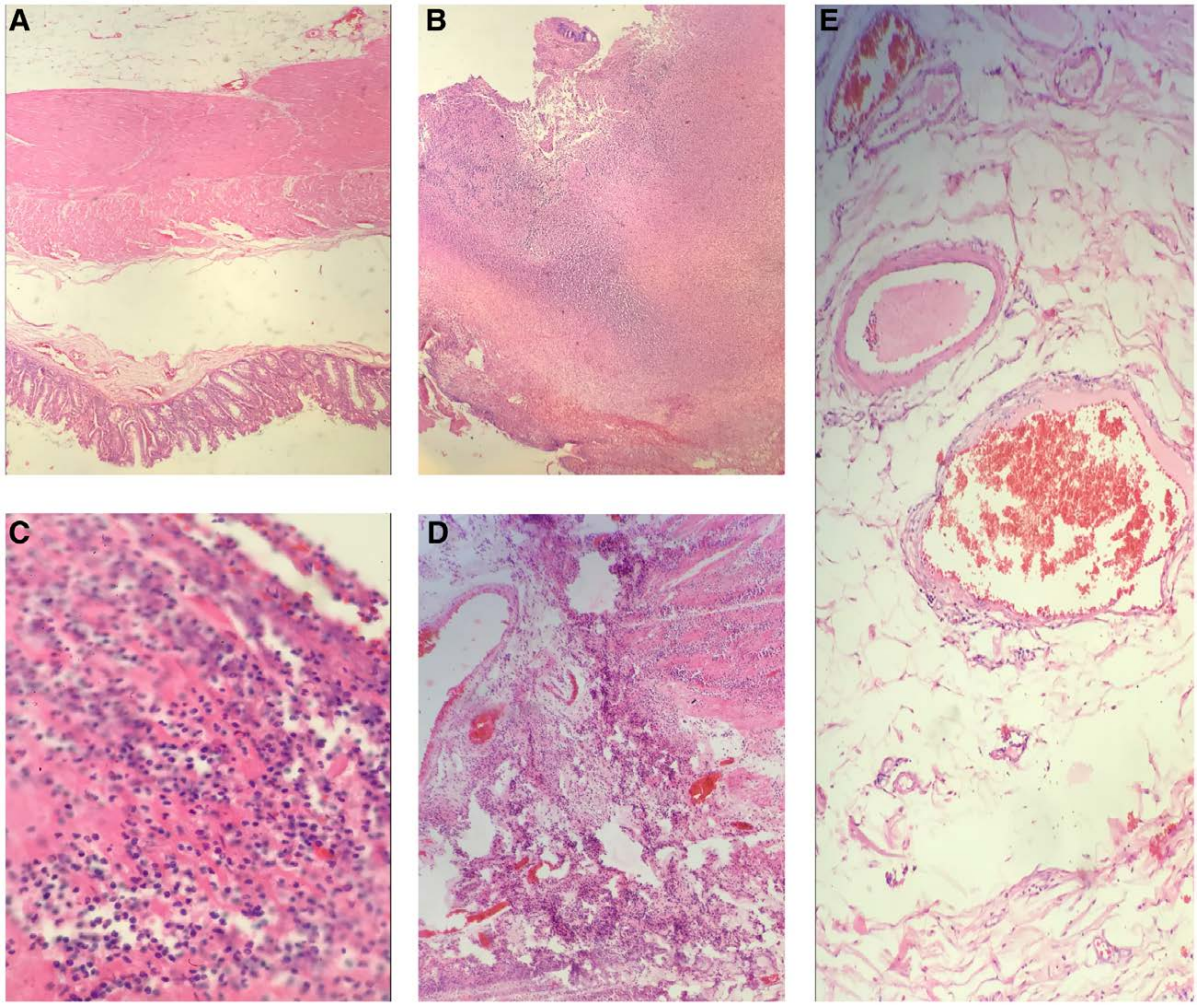
Histological sections evaluated of critical ill patients with COVID 19 (A) Patent intestinal wall. (B) Ischemic and liquefactive necrosis of the intestinal wall. (C) Mixed inflammatory infiltrate: lymphocytic and neutrophilic. (D) Necrosis of the mucosa and intestinal wall, adjacent vessels without thrombi, (E) Patent vessels without thrombi. COVID-19 = coronavirus disease 2019.

The predominant histopathological finding was ischemic and liquefactive necrosis, with mixed inflammatory involvement of lymphocytes and neutrophils. The infiltrate affects the mucosa and leads to necrosis, thereby compromising the thickness of the entire intestinal wall. Vascular thrombosis was ruled out in multiple histological sections evaluated (Fig. 1).

## 4. Discussion

SARS-CoV-2 infection can evolve into multisystemic failure and involve different complications such as acute respiratory distress syndrome (ARDS), acute kidney injury, and venous and arterial thromboembolism.^[6,7]^ The present study included patients with severe pneumonia caused by SARS-CoV-2 and grade IV AGI who required surgical intervention.

In the present study, 93% of patients presented with elevated D-dimer levels and received anticoagulant therapy owing to suspected thrombotic phenomena. Deep vein thrombosis and pulmonary embolism were documented in 13% and 20% of the patients, respectively. However, no mesenteric venous thrombosis was observed in the patients who underwent abdominal CT angiography. Clinical findings that facilitated the identification of suspected AGI cases included abdominal distension, residual gastric volume, constipation, and diarrhea. In this series, all patients underwent surgery, and intestinal biopsies were sent for histopathological analyses, which revealed ischemic and liquefactive necrosis with inflammatory infiltrates in the absence of vascular thrombosis as the cause of intestinal ischemia and necrosis. These findings confirm that the inflammatory process itself is related to the intestinal involvement observed in this cohort of patients. Considering that all patients received anticoagulant therapy and embolic events were objectively ruled out in most of them, it is noteworthy that variables such as lactate and oxygen debt remained high after performing the surgical procedure. The mortality rate was 67%, secondary to MODS, which was higher than that reported in studies including patients with AGI caused by other reasons than SARS-CoV-2 infection.^[3]^ This confirms the complex pathophysiology of the disease.

Drakos et al conducted a study of 218 patients with SARS-CoV-2 infection and AGI. More than 50% of the patients presented with grade III and IV AGI, and worse outcomes, such as longer days of mechanical ventilation provision, longer duration of hospital stay, and increased mortality were reported in 58% of the patients.^[5]^ One of the associations proposed in this study was the relationship between elevated D-dimer levels and AGI, suggesting the presence of a hypercoagulable state that leads to microvascular thrombosis despite the administration of therapeutic anticoagulation. However, since performing autopsies and histopathological analyses for these patients were not possible, this theory could not be confirmed.^[5]^ Different authors consider thrombotic phenomena to be the cause of intestinal perforation and ischemia in patients with SARS-CoV-2 infection,^[5,8–10]^ Observational studies have found that thromboembolic events occur in 21% to 69% of these patients, of which 6.4% have arterial (intestinal and peripheral) thrombosis.^[11,12]^ In this study, histological sections of intestinal tissue collected from patients with grade IV AGI revealed ischemic and liquefactive intestinal necrosis with a severe inflammatory response mediated by neutrophils and lymphocytes in the absence of vascular thrombosis. This finding suggests that inflammation and non-thrombotic phenomena are possible causes of intestinal ischemia and necrosis.

**Figure 2.**
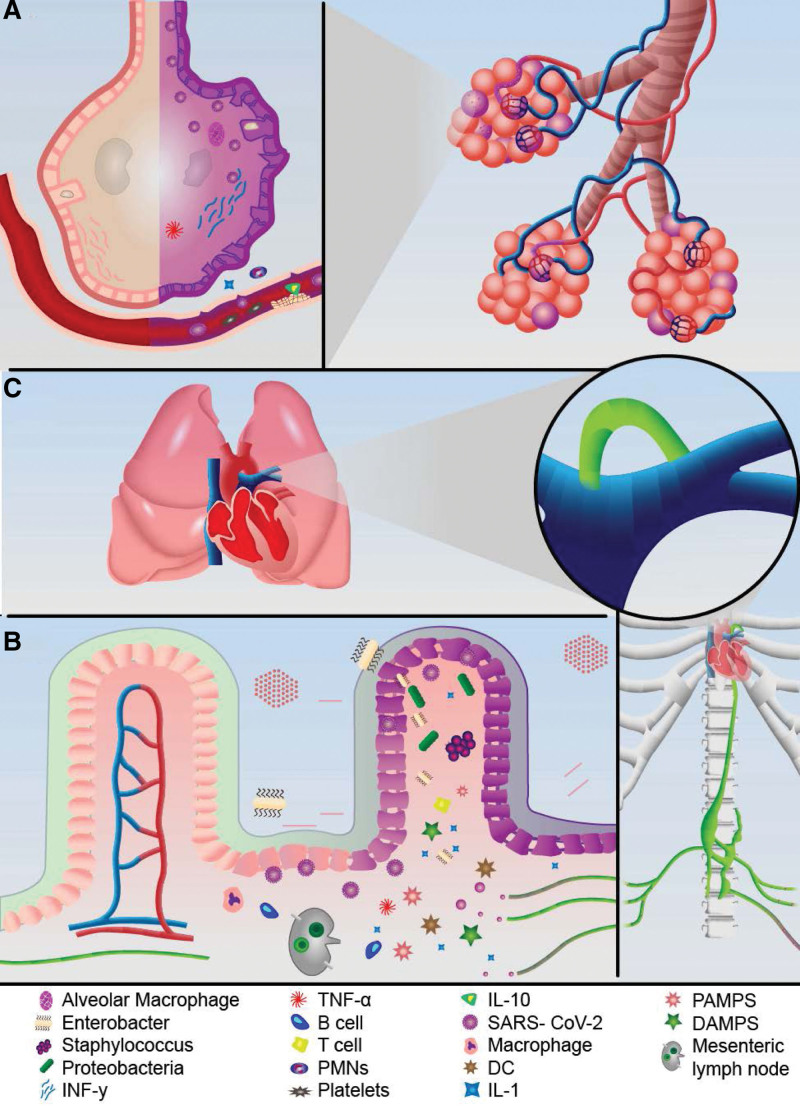
Lungs-gut axis in COVID-19: (A) SARS-CoV-2 infects cells through ACE-2 receptor, located on the surface of alveolar pericytes, endothelial cells, cardiomyocytes, and enterocytes. Initially, there is a direct injury to the cells, generating a systemic inflammatory response mediated by the release of IL 10, IL1, IL6, TNF, and DAMPS. (B) Direct viral injury to gastrointestinal epithelial cells, added to sepsis, generates ischemia with loss of intercellular junctions, necrosis, and loss of the intestinal barrier, favoring bacterial translocation. (C) Humoral and cellular mediators added to bacteria from the gastrointestinal drain to the mesenteric lymph nodes that reach the chyle cistern, later to the thoracic duct and finally through the venous route to the lung, which leads to superinfection and again sepsis. ACE-2 = angiotensin-converting enzyme 2, COVID-19 = coronavirus disease 2019, DAMPS = damage-associated molecular patterns, DC = dendritic cell, IL-10 = interleukin-10, IL-1= interleukin-1, IL6= interleukin-6, PAMPS = pathogen-associated molecular patterns, PMNs = polymorphonuclear cells, SARS-CoV-2 = severe acute respiratory syndrome coronavirus 2, TNF α = tumor necrosis factor alpha.

The intestinal and pulmonary microbiota have been proven to play a fundamental role in critically ill patients. Cross-talk between them (known as the gut–lung axis) has been suggested, in addition to the interaction between endotoxins and microbial metabolites, which causes inflammation in these organs.^[10,13,14]^ In severe SARS-CoV-2, alveolar cell type II damage occurs with a marked inflammatory response mediated by cytokines (IL-1, IL-2, Il-8, IL-120, IL-20), which in the ARDS phase leads to pulmonary edema, hypoxemia, and alveolar and systemic inflammations.^[10,15–17]^ This systemic inflammation triggered by cytokines, damage-associated molecular patterns, and pathogen-associated molecular patterns results in a severe systemic inflammatory response that affects multiple organs, including the colon, which, in addition to the local injury caused by SARS-CoV-2, there is a deceleration of intestinal motility, intestinal secretion, and an increase in blood flow through the stimulation of mu receptors located in the myenteric and submucosal plexus of the gastrointestinal tract, which produces microvascular alterations, oxidative stress, ischemia, and reperfusion with subsequent apoptosis of enterocytes and loss of intercellular junctions with increased intestinal permeability, local inflammation, and bacterial translocation.^[10,13,18–21]^ The lung and colon are connected through mesenteric lymphatic vessels that first drain into the cisterna chyle, then into the thoracic duct, and subsequently into the left subclavian vein, reaching the pulmonary circulation. The lung then becomes the first organ to come into contact with the mesenteric lymph, leading to greater local inflammation and MODS^[10,18–20,22]^ (Fig. 2).

One of the conditions associated with AGI is the use of vasopressors owing to its microvascular hypoperfusion effect on the bowel. In the case of norepinephrine administration, there may be a decrease (up to 77%) in blood flow in the jejunal mucosa, which leads to a state of cellular stress with subsequent AGI.^[23,24]^ In this study, AGI resulting from the use of vasoactive agents was unlikely because 86% of the patients had not received vasoactive agents prior to the onset of gastrointestinal symptoms, and the rest had received low doses of norepinephrine (<0.05 mcg/kg/min). Finally, one of the biomarkers related to mortality proposed by Shoemaker et al was an increase in oxygen debt following the completion of a surgical procedure that included lactate and excess base as predictors of mortality in patients with hemorrhagic shock. This novel predictor can be used to detect multiple organ dysfunction secondary to sepsis.^[25]^

The main limitation of this study was the inclusion of a small number of patients owing to the low incidence of this complication in the ICU. Therefore, performing a prospective multicenter study is necessary to achieve better characterization and identify associated risk factors in this patient population, with the aim of facilitating the management of these patients. Additionally, no histological samples obtained from the lung tissue confirmed inflammatory processes, and no thrombosis was identified in the colon. Therefore, a better histopathological study is required to understand dysbiosis as a possible cause of lung and colon inflammation, which worsens ARDS and increases the risk of intestinal ischemia and necrosis.

## 5. Conclusion

Severe AGI in patients with SARS-CoV-2 infection poses a diagnostic challenge in the ICU, however, it may be associated with an inflammatory response in the gastrointestinal tract that may lead to ischemia and intestinal perforation. More analytical studies are required to identify, understand, and identify risk factors for this pathology.

## Acknowledgments

Fundación Clínica Shaio.

## Author contributions:

**Conceptualization:** Buitrago-Bernal Ricardo, Pérez-Garzón Michel, Forero-Delgadillo Alex; Pérez-Garzón Michel, Robayo-Amortegui Henry.

**Data curation:** Robayo-Amortegui Henry, Forero-Delgadillo Alex, Muñoz-Claros Conny, Bayona-Solano Andrea, Perez-Garzón Michel.

**Formal analysis:** Robayo-Amortegui Henry, Pérez-Garzón Michel, Poveda-Henao Claudia.

**Methodology:** Robayo-Amortegui Henry, Pérez-Garzón Michel, Poveda-Henao Claudia, Muñoz-Claros Conny.

**Supervision:** Buitrago-Bernal Ricardo, Pérez-Garzón Michel, Poveda-Henao Claudia.

**Validation:** Orozco Carlos, Poveda- Pérez-Garzón Michel, Poveda-Henao Claudia.

**Writing – original draft**: Robayo-Amortegui Henry, Forero-Delgadillo Alex, Muñoz-Claros Conny, Bayona-Solano Andrea, Perez-Garzón Michel, Poveda-Henao Claudia.
